# Blood cancer prediction model based on deep learning technique

**DOI:** 10.1038/s41598-024-84475-0

**Published:** 2025-01-13

**Authors:** Amr I. Shehta, Mona Nasr, Alaa El Din M. El Ghazali

**Affiliations:** 1 Department of Information System, Faculty of Computers and Artificial Intelligence, Helwan University, Cairo, Egypt; 2 Department of Computer and Information Systems, Sadat Academy for Management Sciences, Cairo, Egypt

**Keywords:** Blood cancer, Medical deep learning, Classification, ResNetRS50, RegNetX016, VGG19, Convnext, Cancer, Diseases, Health care, Medical research, Oncology, Risk factors, Mathematics and computing

## Abstract

Blood cancer is among the critical health concerns among people around the world and normally emanates from genetic and environmental issues. Early detection becomes essential, as the rate of death associated with it is high, to ensure that the rate of treatment success is up, and mortality reduced. This paper focuses on improving blood cancer diagnosis using advanced deep learning techniques like ResNetRS50, RegNetX016, AlexNet, Convnext, EfficientNet, Inception_V3, Xception, and VGG19. Among the models assessed, ResNetRS50 had better accuracy and speed with minimal error rates compared with other state-of-the-arts. This work will exploit the power of ResNetRS50 in contributing to early detection and reducing bad outcomes for blood cancer patients. Blood cancer is currently one of the deadliest diseases worldwide, resulting from a combination of genetic and non-genetic factors. It stands as a leading cause of cancer-related deaths in both developed and developing nations. Early detection of cancer is pivotal in reducing mortality rates, as it increases the likelihood of successful treatment and potential cure. The objective is to decrease mortality rates through early diagnosis of blood cancer, thus offering individuals a better chance of survival from this disease.

## Introduction

Blood cancer, more commonly termed hematological malignancy, originates from bone marrow or cells with the ability to form blood. Such a cell produces red and white blood cells and platelets. The latter then show a transformed growth through cancer, which could hamper the normal functions of the body. In^1^, There are many types of blood cancers such as Leukemia: The white blood cells production in an uncontrolled fashion. Leukemia is a group of blood cancers that predominantly affect the white blood cells of the body. Leukemia may occur when some blood cells do not develop properly. It is diagnosed mainly in people over age 55, but it also is very common in children below 15. Over 60,000 US citizens are diagnosed with leukemia each year^2^. Symptoms of leukemia-which will, of course, vary from person to person-may include infections that are frequent or severe, bleeding or bruising easily, repeated nosebleeds, and small red spots in the skin. in^3^. First, it can be classified according to the kind of WBCs affected. In this category, there are two kinds. The first is Lymphocytic leukemia-that affects the lymphoid cells or lymphocytes, from where lymphatic tissues are formed. While the second is myelogenous leukemia, which affects myeloid cells that produce RBC, WBC, and blood platelets^3^. Lymphoma: This is a type of cancer starting from the lymph nodes and other parts of the lymphatic system, and Multiple myeloma: This is a form of blood cancer that represents a malignancy of plasma cells, which are a type of white blood cells.

Blood cancer can have serious health and quality-of-life effects on the individual. Symptoms include, but are not limited to fatigue, fever, weight loss, tendency to bleed frequently, or bruises that occur easily, along with frequent infections, depending on the type and stage of the disease. Treatment may be successful with early detection and proper diagnosis. Seek medical attention if any of these symptoms are encountered.

These are a few deep learning methods that can solve problems beyond complex for the human brain to handle with utter success and consistently. A proposed approach of supervised machine learning for the prediction of blood cancer diseases, it has used a leukemia microarray gene dataset comprising 22,283 genes. Some of the other related works solve the problem of imbalanced and high-dimensional datasets by incorporating techniques such as ADASYN resampling and Chi-squared feature selection. Here, synthetic data with ADASYN is created, balancing the dataset regarding each target class; further selecting the best 22,283 characteristics for training learning models using Chi2^4^. The detection of blood cancer in its early stage is crucial for its successful treatment. Early detection of cancer in the blood system is, therefore, an important factor that ensures successful treatment and recovery. Early signs and risk factors of the disease enable the patient to take early diagnosis, which makes treatment far more effective. On the contrary, the survival rates decrease whenever there is any delay in detection because the disease continues to progress without any hindrance with the passage of time.

In this research, our work contributions are as follows:

- The research demonstrates how stain-specific information enhances cancer detection accuracy in deep learning for medical imaging.
- It combines advanced architectures with stain deconvolution techniques, expanding diagnostic imaging applications.
- The work aims to push innovation in AI-assisted cancer diagnosis while maintaining ethical standards and patient care.

The rest of the paper is structured as follows. Section 2 discusses related work. Section 3 provides definition of blood cancer, Sect. 4 provides proposed Methodology of research, Sect. 5 provides proposed model, Sect. 6 provides experimental result, Sect. 7 provides discussion, Sect. 8 provide clinical relevance, Sect. 9 provides challenges and future work to consider. Section 10 includes our conclusion.

## Related work

In^5^, In this paper, a review of 1,732 pictures of bone marrow to train a CNN by introducing new deep learning algorithms and using raw photos with no pre-processing done on them, the authors were able to put together an end-to-end leukemia detection system. The algorithm performed clever imitation of the process of the hematologist, in that it identified and excluded uncountable and smashed cells and classified and counted the residual ones for diagnosis. The system’s overall performance in WBC classification gave an accuracy of 82.93%, a precision of 86.07%, and an F1 score of 82.02%. This technique had an overall performance for acute lymphoid leukemia diagnosis with a sensitivity of 89%, an accuracy of 86%, and a specificity of 95%. The method also did well in detecting lymphoma and neuroblastoma dissemination to the bone marrow, with an average accuracy of 82.93%. This study was among the first that employed a wider range of cell types for the diagnosis of leukemia and showed relatively good performance in real clinical practice.

In^1^, a machine learning used to track the progression of the disease in children undergoing treatment for ALL. Although several prognostic biomarkers are known at the time of diagnosis, the single most important independent prognostic factor is the response of the leukemia to induction chemotherapy. Bone marrow samples used for diagnosis and treatment were stained with an ALL-discriminating antibody cocktail and analyzed by imaging flow cytometry. By using only features from bright-field and dark-field cell images-without fluorescent markers-a deep learning algorithm achieved classification of Acute Lymphoblastic Leukemia cells with an accuracy of over 88%. This antibody-free single-cell technique is fast, inexpensive, and can be performed with basic cytometers lacking lasers, thus enabling automated point-of-care testing to identify delayed early responders.

In^6^, Microscopic image analysis for automatic leukemia detection was discussed in^6^. this study was performed with elaborate pre-processing to overcome the main difficulties during the segmentation phase and proposed an efficient three-stage filtration algorithm to refine the segmentation results. It also extracted sixteen robust features from the images by following the procedures of hematological experts. This greatly enhanced the capabilities of the classifiers in distinguishing the leukemic cells in the microscopic images. During the classification study, the traditional machine learning classifiers involved were the artificial neural network and the support vector machine. They both shared a specificity level of 95.31%, while the support vector machine had levels like that of the sensitivity for the artificial neural network.

In^7^, a CNN architecture was proposed to classify the blood slides as Acute Myeloid Leukemia, Acute Lymphoblastic Leukemia, and Healthy Blood Slide. The experiments were done using 16 datasets of 2,415 images, resulted in the accuracy rate of 97.18% and a precision score of 97.23%.

In^8^, multi-class cancer detection in a digital healthcare paradigm, based on distributed fog and cloud networks, has thus obtained remarkable enhancement in recent years. The paradigm enables the collection and training of cancer data on various computing nodes to optimally detect cancer with their respective classes.

In^9^, This research reviews and evaluates key studies on deep learning (DL) models in the omics field, aiming to showcase their potential and highlight challenges. It systematically surveys literature from 2018 to 2022 across four databases (IEEE Xplore, Web of Science, ScienceDirect, PubMed), resulting in 65 relevant articles. Of these, 42 focus on clinical applications of DL, 16 are reviews on single- and multi-omics data, and 7 cover comparative analysis and guidelines. The study addresses obstacles such as DL model challenges, preprocessing, and validation, offering insights for practitioners into the role of DL in omics data analysis.

In^10^, The main goal of this study is to review and evaluate the best research on AI methods in the omics field. It highlights the potential of AI in omics data analysis and identifies key challenges that need addressing. The study uses a systematic approach to search for relevant articles, focusing on clinical applications, guidelines, comparative studies, and reviews. Challenges like AI models, preprocessing, datasets, and validation are discussed. This study offers new insights into the intersection of AI and omics, providing practitioners with a comprehensive understanding of AI’s role in omics data processing.

## Blood cancer definition

Some causes of leukemia include radiation exposure, family history, and exposure to certain chemicals. Most commonly, leukemia can be categorized based on the rate at which it progresses, and the type of cell involved. In many cases, the disease’s progression will initially classify leukemia into either one of two categories: acute leukemia and chronic leukemia^11^.

###  Types of leukemia

They are mainly categorized depending upon the type of WBCs which is being affected. According to this category, there are two classes. First one is the Lymphocytic leukemia, through which lymphoid cells or lymphocytes, from which lymphatic tissues are formed gets affected. The second one is the Myelogenous leukemia which affects the myeloid cells, responsible for forming the RBCs, WBCs, and platelets in the blood.

The second type of leukemia classification is according to the speed at which it affects the human body; for instance, acute leukemia. In acute leukemia, the cancerous cells are immature blasts. These cells grow and develop very fast and cannot perform their normal functions. Patients suffering from acute leukemia need vigorous and fast treatments^3^.

**Fig. 1:**
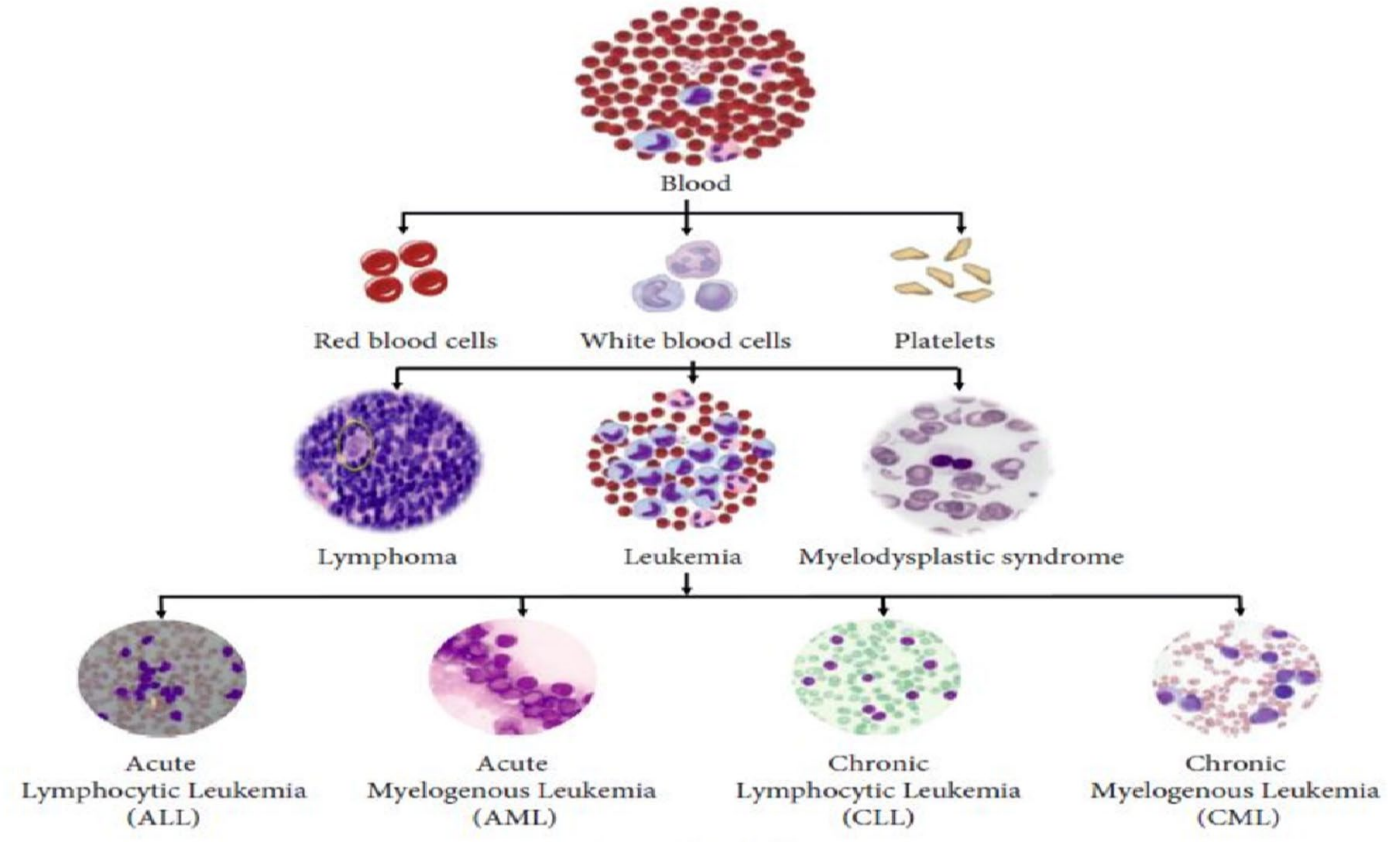
Leukemia types^10^.

Doctors in the US diagnose about 90,000 people with lymphoma every year. Lymphoma arises from a white blood cell known as lymphocytes. The two major types of lymphocytes, which include B cells and T cells, each have malignant cells that grow abnormally. Lymph tissue is located throughout the patient’s body; hence, lymphoma can start developing almost anywhere^6^. Figure 1 explains the types of leukemia.

Aside from those categorizations, leukemia has a multitude of subtypes. These subtypes include^12^:

- Acute lymphocytic leukemia, mostly occurring among children.
- Acute myelogenous leukemia, common in adults and may affect children also.
- Chronic lymphocytic leukemia is the most common chronic leukemia in adults.
- Chronic myelogenous leukemia (CML), It could go undetected for many weeks, even months. By the time it is, the leukemia could have reached that gradual quick spreading phase^13^.

###  Leukemia treatments

There can be no absolute screen for leukemia. In contrast, it may be manifested in a normal blood test. If diagnosed, there are several treatment options including^14^:

- *Observation*: In few cases, leukemia may not be symptomatic that mean a patient can go for observation. In these instances, there might be no treatment necessary at all until they have symptoms, or their current symptoms worsen. The doctors will continue to monitor the state during this time.
- *Drugs*: Several drugs are effective in treating leukemia:
- *Chemotherapy*: This involves killing the cancer cells^15^.
  - *Immunotherapy*: This treatment method is all about making the patient’s immune system more vigilant in combating against the cancer cells^16^.
  - *Targeted therapy*: targets specific genes in the body to fight cancer.
- *CAR T-cell therapy*: This is an immunotherapy treatment that uses a patient’s own immune cells to fight their cancer. Doctors May Slingshot Challenger for Parts in Leukemia Treatments^17^.
- *Stem cell transplant*: In this course of treatment, the patient’s blood or bone marrow with defective cells are replaced with healthy ones. These might derive from a healthy donor—even possibly the patient’s own body. Patients usually receive chemotherapy before reaching the point where a stem-cell transplant is considered^18^.

###  Selection deep learning techniques

They destroy either the cancer cells or they stop these to divide and grow new cancer cells to replace the dead ones^2,19^ via a Selection Deep Learning Techniques. This means the following research will build a classification model using deep learning. The deep learning algorithm holds potential for predicting and diagnosing the blood cancer. Since our main goal in deep learning is to find relationships between specific characteristics, the function of CNN in this study is to classify the data. Through the ResNetRS50, RegNetX016, AlexNet, EfficientNet, Inception_v3, Xception and VGG19 (Convnext) algorithm. We have used commonly referenced models in this research including ResNetRS50, RegNetX016, EfficientNet and Convnext.

## Methodology of research

The methodology section gives detailed description of tool, techniques and steps taken while Building &Training ResNetRS50 and RegNetX016 deep learning model for cancer detection. The study includes the sub segments:

### Data collection and preprocessing

- *Data collection*: This research has been taken the dataset from the second sources more specifically from Kaggle^20^. Leukemia (15,135 images).
- *Data preprocessing*: The dataset was preprocessed by resizing all the images to a common resolution (224 × 224 pixels), denoised and augmented using techniques like rotation, horizontal flipping and contrast adjustments.
  - *Resizing*: All images have been resized to the same size so that they are compatible with the neural network architecture. Images are resized to 224 × 224 pixels. A very common size for many deep learning models, mainly applied in models coming from architectures like ResNet and VGG.
  - *Denoising*: It would be in the purview of such techniques as Gaussian blurring, median filtering, or even wavelet denoising that some of the noise or other kinds of artifacts affecting the quality of learning meaningful patterns in the model could be gotten rid of.
  - *Augmentation*: This would increase variation in training data and thereby enhance the generalizing capability of the model.
  - *Rotation*: There will be random rotations of images to represent different viewpoints.
  - *Horizontal Flipping*: Images are flipped horizontally to create new yet realistic variations.
  - *Contrast Stretching*: This enhances the contrast between different image intensities to simulate various lighting conditions.
  - *Combination*: All these steps for preprocessing make the dataset robust and good for deep learning training. It will help reduce overfitting and improve performance, giving more capability to the model to handle new unseen data.

### Model selection and architecture

- *Model selection*: We finally chose to ResNetRS50 and RegNetX016 for their reputation in medical image analysis. These models are known for their ability to pick up fine details from images.
- *Model architecture*: To make predictions with them, we needed to add a few custom top layers (fully connected layers + SoftMax output layer) on both models. Dropout and L1/L2 regularization were used for generalization purposes.

### Training and evaluation

- *Dataset*: Chinese National Medical Centre “CNMC” is an open-access dataset of blood cancer images. This dataset was developed by a research team of the Chinese National Medical Centre and includes many leukemia, lymphoma, and multiple myeloma images. This dataset contains an appreciable number of images in all diversities of the various cases that involve blood cancers. It has pictures of different types of blood cancers, which can have the model developed in a way that will classify or diagnose variants of such diseases with efficiency. The C-NMC dataset pictures are clear, hence suitable for training and testing of deep learning models. Each image is labeled to show the specific form of blood cancer. The C-NMC dataset source code is open, hence any resource a researcher may seek is made available from the dataset toward any number of studies or projects needed. Potential use for the C-NMC dataset: The dataset can be used to train and evaluate deep learning models for the classification and diagnosis of blood cancers. This will provide the opportunity to try new diagnostic and detection methodologies of the blood cancer, for instance, systems of computer-aided diagnosis. The data set can be used in developing tools that can assist healthcare professionals in making more accurate and informed decisions regarding the diagnosis and treatment of blood cancer. The C-NMC dataset forms the cornerstone for any researcher and clinician working within the framework of blood cancer. It is important because it will, through the quantity and the difference in the images provided, help in improving knowledge about this multi-factorial sickness and enhance patient outcomes^21^.
- *Training procedure*: Adam is one of the most popular optimization algorithms used across deep learning, particularly in the training of neural networks. Adam adapts the learning rate for each parameter in a different way; hence, it can adapt to the characteristics of the data and the optimization landscape. Adam is computationally efficient and easy to implement. Accordingly, Adam is very computationally efficient and converges rather sooner, hence recommended for large-scale deep learning tasks. Due to that fact, Adam is relatively robust concerning different learning rates and hyperparameter settings and thus was convenient for use. Being wide-spread applied, it was used in training CNNs, RNNs, and GANs. Adamax optimizer was used to train the models with learning rate set at 0.001. The training dataset was trained for 40 epochs with a batch size of 40. The training was divided into 70% training and 30% validation set.
- *Evaluation metrics*: The model performance was evaluated in terms of the following metrics: true positive (TP), false negative (FN), generating confusion matrix, accuracy, precision (positive predictive value- PPV), recall (sensitivity or hit rate) and F1 score for binary classification to make sure it does not have a certain biasness towards some class labels scores. The early stopping was reduced using the validation loss.

### Hyperparameter tuning

- *Learning rate*: Hyperparameter tuning involved exploring different learning rates (0.001, 0.01, 0.0001) to identify the optimal rate for each model.
- *Batch size*: Various batch sizes (16, 40, 64) were tested to assess their impact on model convergence.

### Callback implementation

- *Early stopping*: During training, an early stopping callback was utilized with a patience of 5 epochs. This callback function was implemented to halt the training process if there was no improvement in the validation loss.
- *Learning rate adjustment*: A learning rate reduction callback was integrated into the training process. This callback function was designed to decrease the learning rate by a factor of 0.5 if the validation loss remained stagnant for 2 consecutive epochs.

### Documentation and reporting

- *Comprehensive documentation*: A documentation framework was established to cover data preprocessing, model architecture, training procedures, and evaluation methods.
- *Reporting*: The documentation includes detailed performance reports, confusion matrices, and examples of model predictions on test data.

### Validation and testing

- The models were rigorously validated on a distinct test dataset, and the results of these validation tests were exhaustively reported. Insights into the generalization capabilities of the models were provided.
- *Testing*: A separate test dataset was used to assess the model generalization. Results showed an accuracy of 88% on unseen data.

## Proposed model

**Fig. 2:**
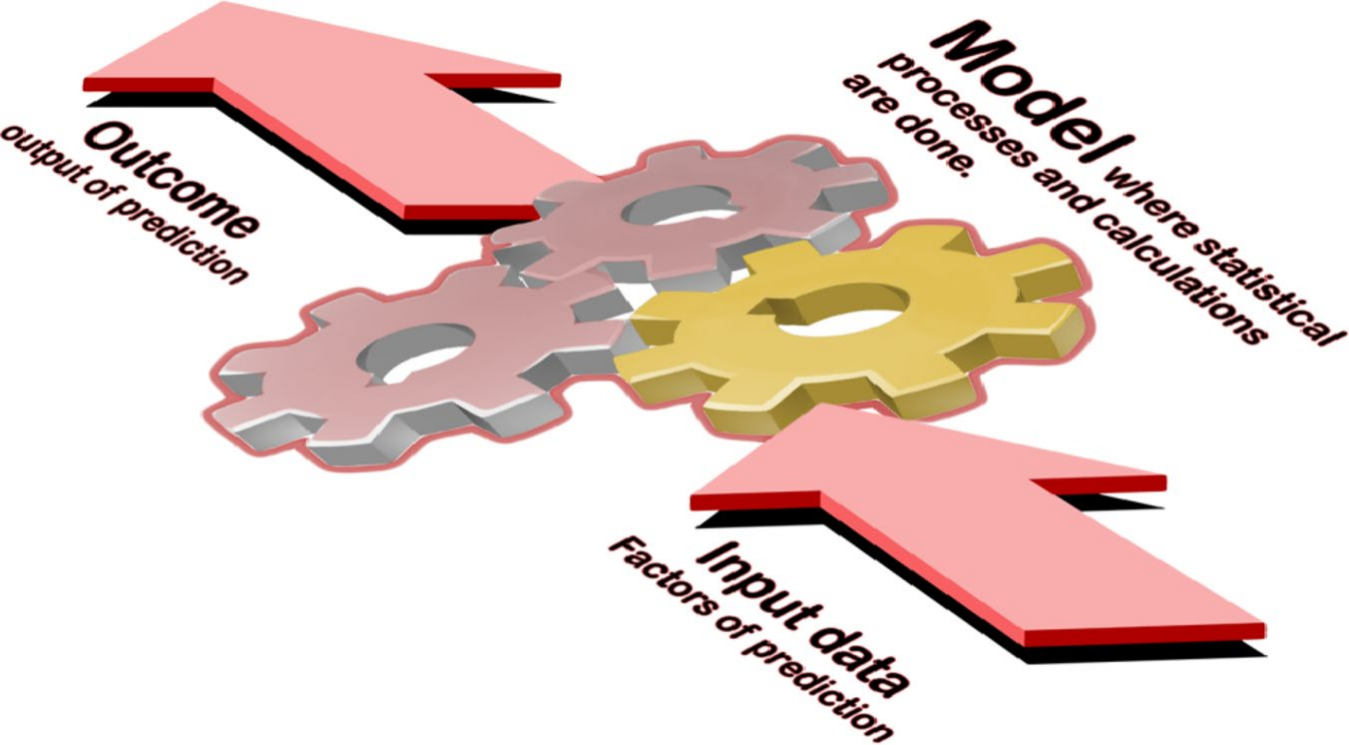
Cycle of predictive modeling.

Predictive modeling is a process of deep learning and mathematical probability to forecast outcomes or trends based on historical data. t involves the step-by-step procedure of collection and preparation, model building, Model Evaluation, and Deployment. The model has multiple predictive factors that might also be referred to as images in arriving at the output of the model. Predicting systems, as would be seen, range from meteorology and weather forecasting through to medical applications. As such, the concept can schematically be represented in Fig. 2.

Once a sample of data related to the wanted output is gathered, data cleaning and selection stages follow to refine the data set. After that, predictive attributes are assigned to the data. Then, a mathematical method of deriving the output based on the assigned attributes must be formulated.

### Analysis of blood cancer prediction model

Blood cancer ranks as the most frequently diagnosed cancer and stands as the second leading cause of cancer-related deaths among them. Given its prevalence and impact, addressing this issue becomes a high-priority concern. It is imperative for researchers to exert maximum effort in predicting the disease early to enhance the chances of recovery.

**Fig. 3:**
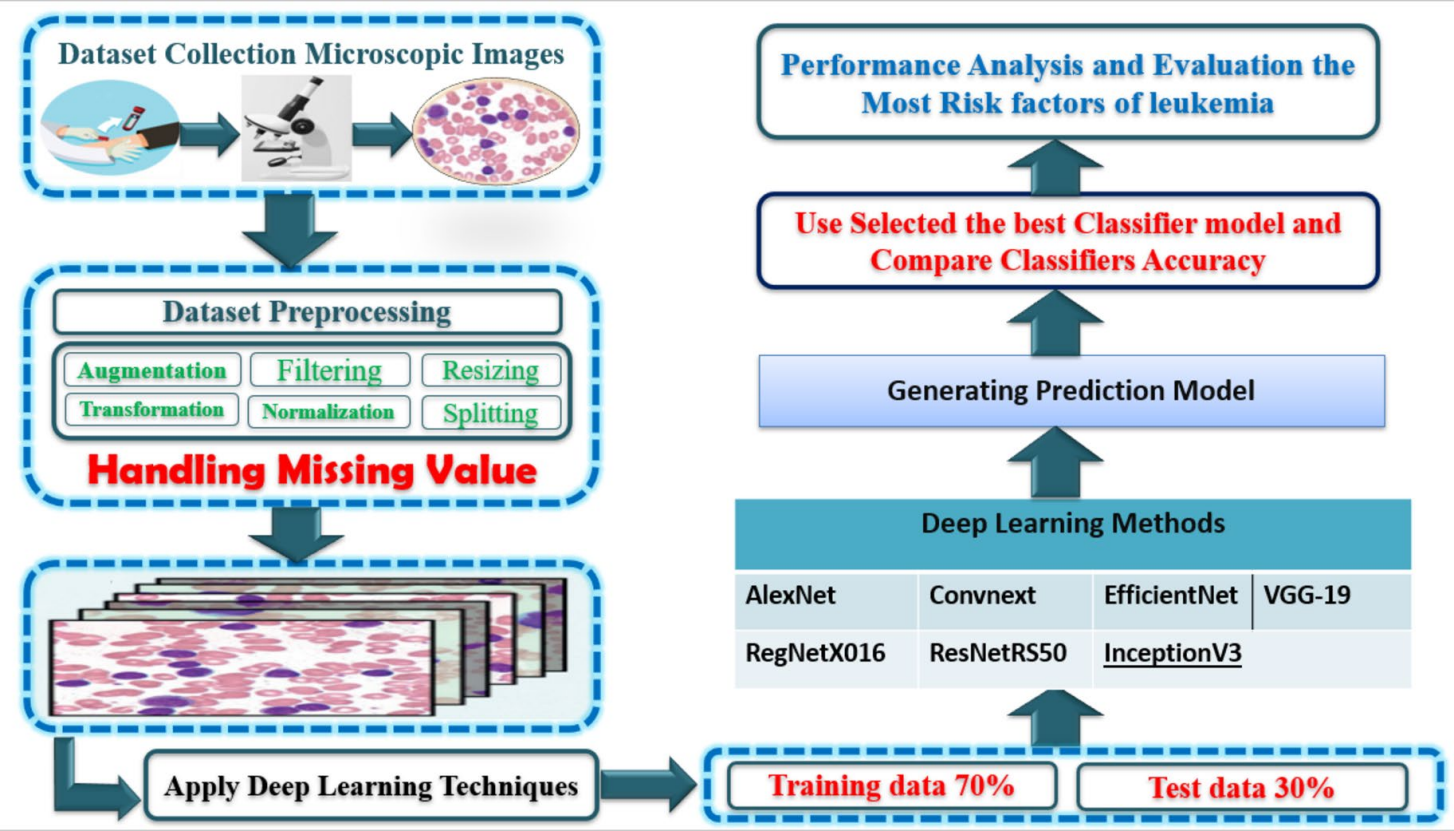
Architecture of blood cancer prediction model.

The stages of this model’s architecture include the following: (see Fig. 3)

The model provides an in-depth understanding of the tools, techniques, and procedures used to develop and train deep learning models for cancer detection.

## Experimental results

In this section, we provide a comparative analysis of various models used in our study, highlighting their architectural differences and corresponding accuracy results:

1. *ResNetRS50*: is a deep learning architecture with a considerable focus on computer vision tasks, mainly image classification and object detection. Indeed, ResNet families of neural networks have the capability to handle very deep networks without having any vanishing gradient problem.
  *Architecture*: The deep Architecture provides the ability of the network to learn complex features and relationships in image data. ResNet (Residual Neural Network) with 50 layers which is represented in Fig. 4.
  *Accuracy*: Achieved an accuracy of **97%.**
  *Comments*: ResNetRS50 exhibited strong performance, making it a competitive choice in our study.
  **Fig. 4:**
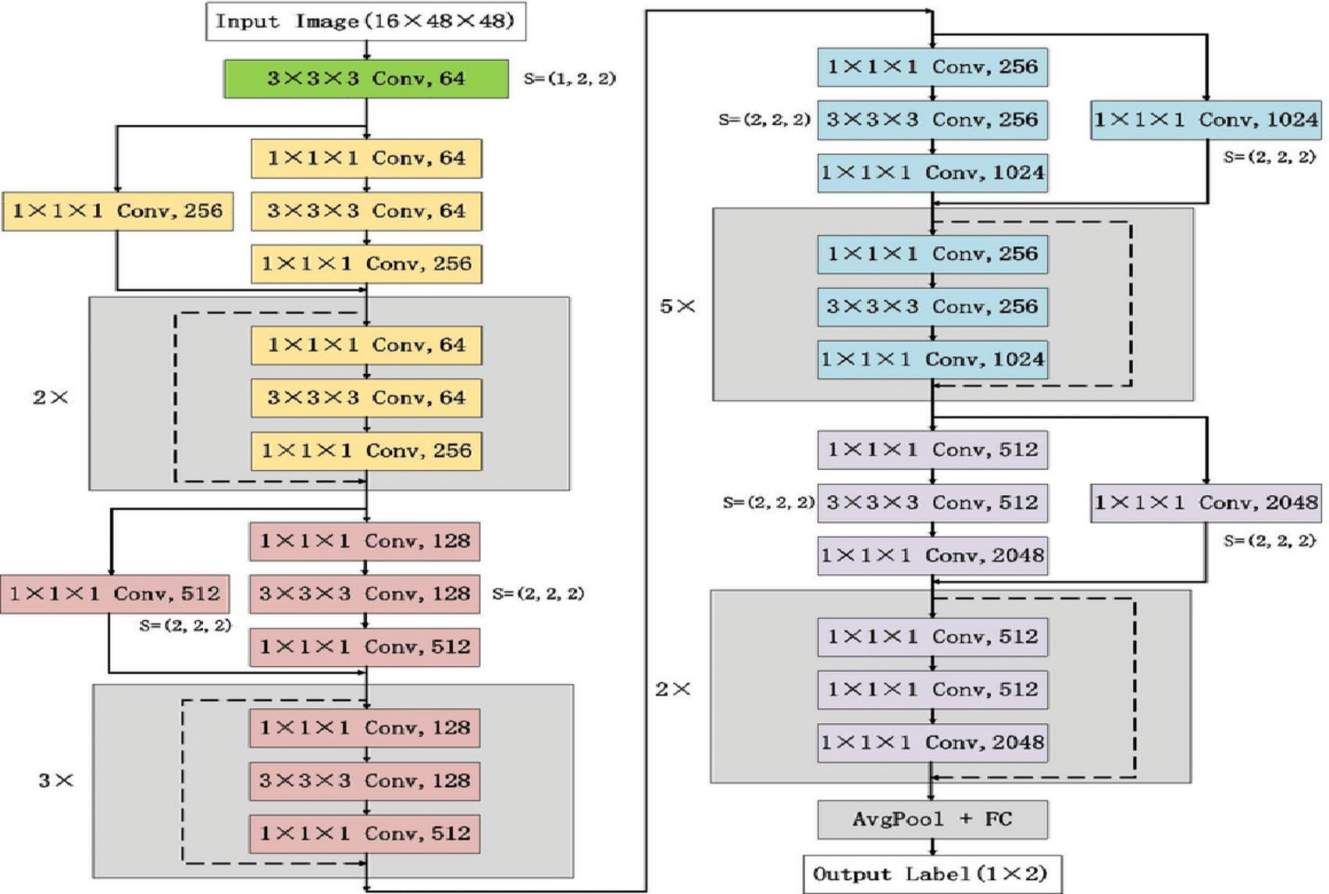
Architecture of ResNetRS50.
2. *RegNetX016*: represents a deep learning architecture created to be scalable and efficient. The RegNet family of networks works on a mathematical formulation that allows them systematically to explore different aspects of network architecture. Accordingly, RegNetX016 can be scaled up or down in size and depth to adapt to different computational resources or even task requirements. Lightweight yet effective architecture-a balance of performance and resource utilization. RegNetX016 follows a specific design to avoid over-fitting using the regularization technique and give better generalization. Applications of RegNetX016 may be done for image classification tasks, such as object recognition, scene classification, and medical image analysis. By properly representing features, the network can be used as a backbone network for object detection algorithms in which objects are detected and localized within images. They can adapt RegNetX016 to be used in image segmentation problems where the general goal is always to assign a semantic label for each pixel in an image. This skill of scaling the architecture to other sizes and depths supports applications that suit a range of computational resources and tasks with differing needs. Therefore, RegNetX016 is computationally efficient and intended to work on resource-constrained devices. Results on various benchmarks showed the strong performance of RegNetX016 competitive to other state-of-the-art architectures.
  *Architecture*: RegNetX with a configuration of 016 which is represented in Fig. 5.
  *Accuracy*: Achieved an accuracy of 96.6%.
  *Comments*: RegNetX016 outperformed other models in terms of accuracy, making it a top performer.
  **Fig. 5:**
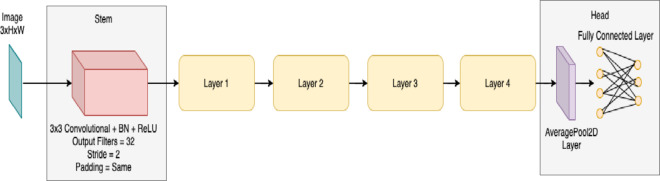
Architecture of RegNetX.
3. *AlexNet*: it was one of the first deep convolutional neural networks and had a total of eight layers-five convolutional, two fully connected, and finally a SoftMax layer-which allowed it to learn complex features from image data because of such great depth.
  *Architecture*: AlexNet, a classic deep convolutional neural network which is represented in Fig. 6.
  *Accuracy*: Achieved an accuracy of **87.9%.**
  *Comments*: While AlexNet is well-known, it exhibited slightly lower accuracy compared to other models.
  **Fig. 6:**
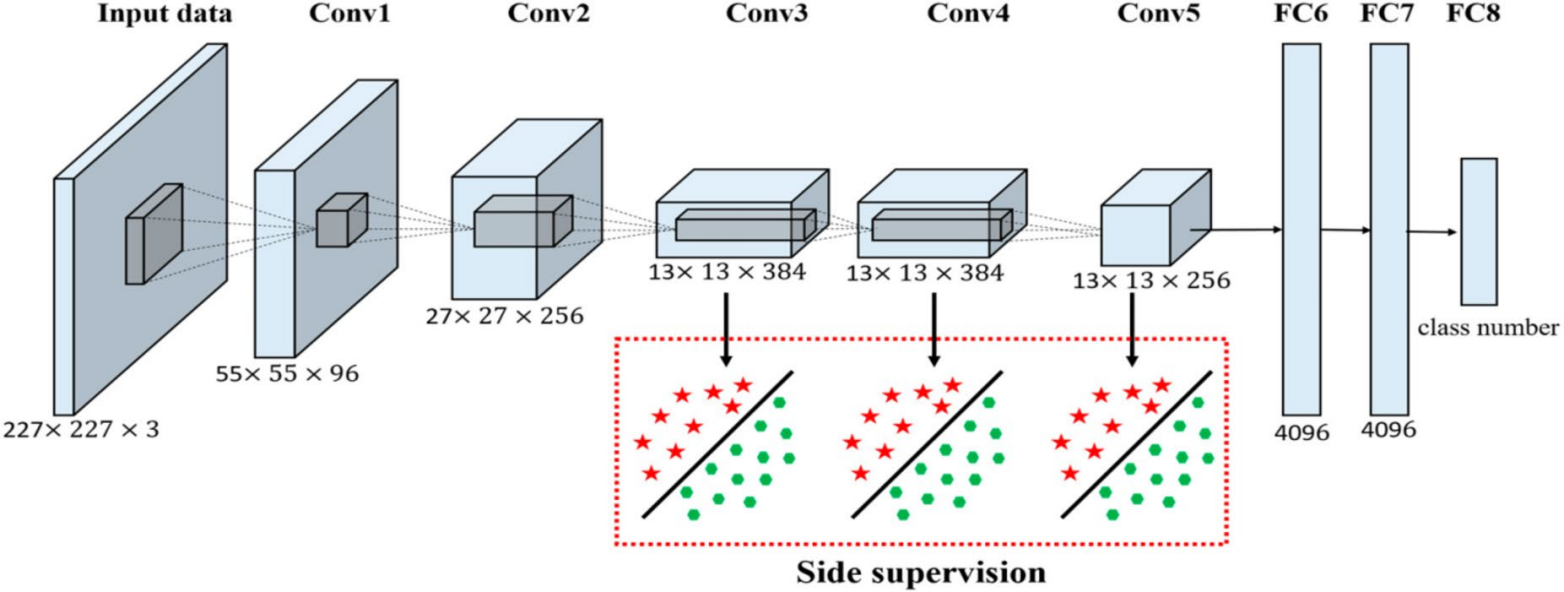
Architecture of AlexNet.
4. *Convnext*: This is the state-of-the-art CNN architecture, proposed for image classification. ConvNext introduces re-scaled convolutions that apply a scaling factor on the output channels of each convolutional layer. This can help the network to be more efficient and accurate. Unlike in traditional CNNs, the layer normalization of ConvNext is applied before the activation function, which may help improve training stability and enhance its performance. Advantages it possesses are high performance, efficiency, and flexibility.
  *Architecture*: Convnext, a custom architecture designed for the task which is represented in Fig. 7.
  *Accuracy*: Achieved an accuracy of **94.2%.**
  *Comments*: Convnext showed competitive accuracy, indicating its suitability for the task.
  **Fig. 7:**
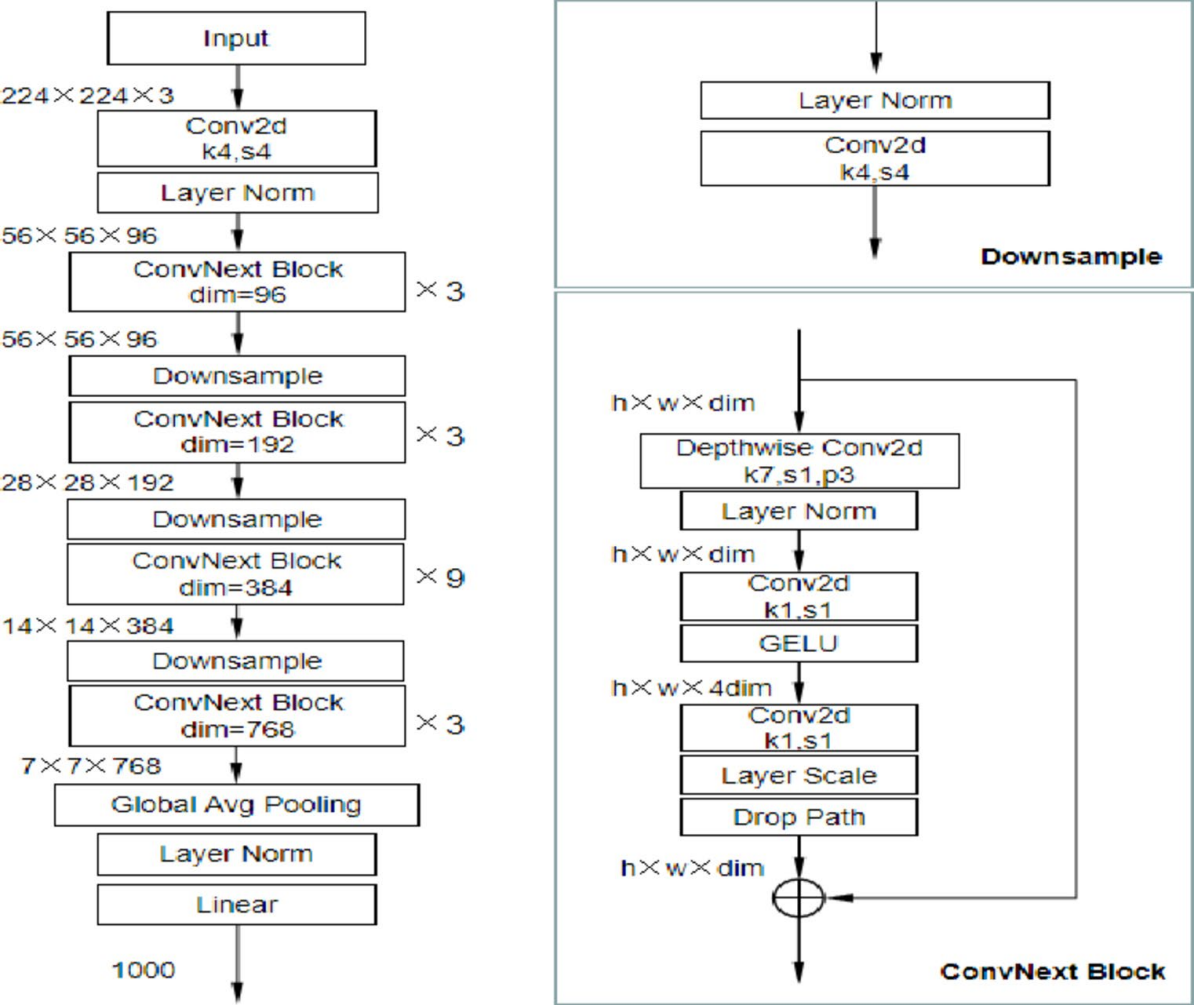
The architecture of Convnext.
5. *EfficientNet*: It is a family of convolutional neural network models designed for higher accuracy, and this is achieved with substantially fewer parameters and at lower computational cost compared to other models. EfficientNet can be realized using the compound scaling method to scale up the width, depth, and resolution of the network uniformly. Advantages of EfficientNet: high accuracy, efficiency, and scalability.
  *Architecture*: EfficientNet, a family of models with efficient scaling which is represented in Fig. 8.
  *Accuracy*: Achieved an accuracy of **93.0%.**
  *Comments*: EfficientNet demonstrated good accuracy while being computationally efficient.
  **Fig. 8:**
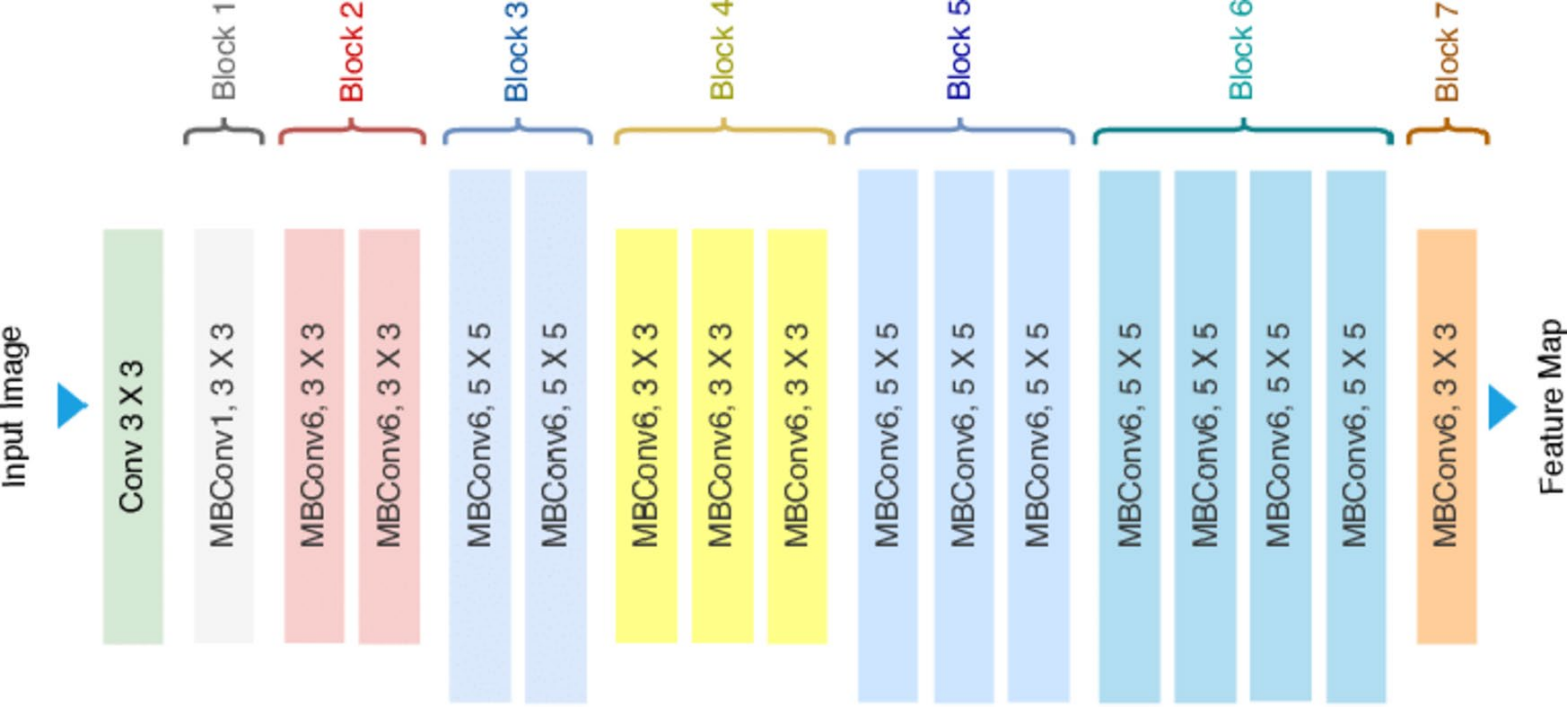
The architecture of EfficientNet.
6. *Inception_v3*: it is an architecture of the convolutional neural network. The family of inception models is very famous for their moderately computational resource efficiency coupled with their capabilities of capturing intricate features in an image. It has some advantages: efficient, accurate, and scalable.
  *Architecture*: Inception_v3, known for its Inception modules which is represented in Fig. 9.
  *Accuracy*: Achieved an accuracy of **92.7%.**
  *Comments*: Inception_v3 showed decent accuracy but was slightly outperformed by some other models.
  **Fig. 9:**
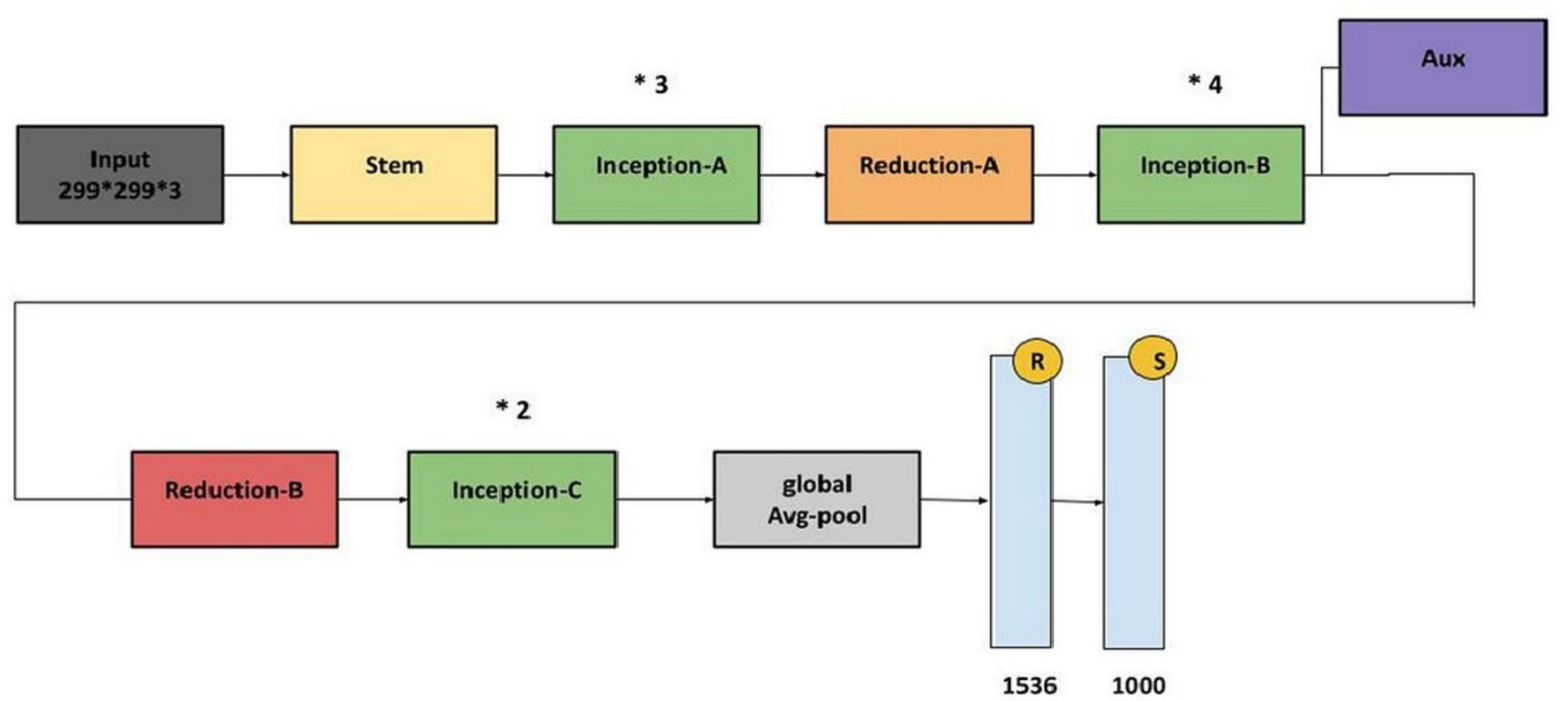
The architecture of InceptionV3.
7. *Xception*: Xception is a deep CNN architecture. It is a modification of the Inception_v3 model. It differs from Inception_v3, where it changes all Inception modules with depthwise separable convolutions. Residual connections are also included in Xception models, as in ResNet, which helps to regularize the training and avoids the problem of vanishing gradient. Advantages of Xception Efficient Accurate Scalable.
  *Architecture*: Xception, an extension of the Inception architecture which is represented in Fig. 10.
  *Accuracy*: Achieved an accuracy of 91%.
  *Comments*: Xception exhibited good performance, though its accuracy was slightly lower compared to top-performing models.
  **Fig. 10:**
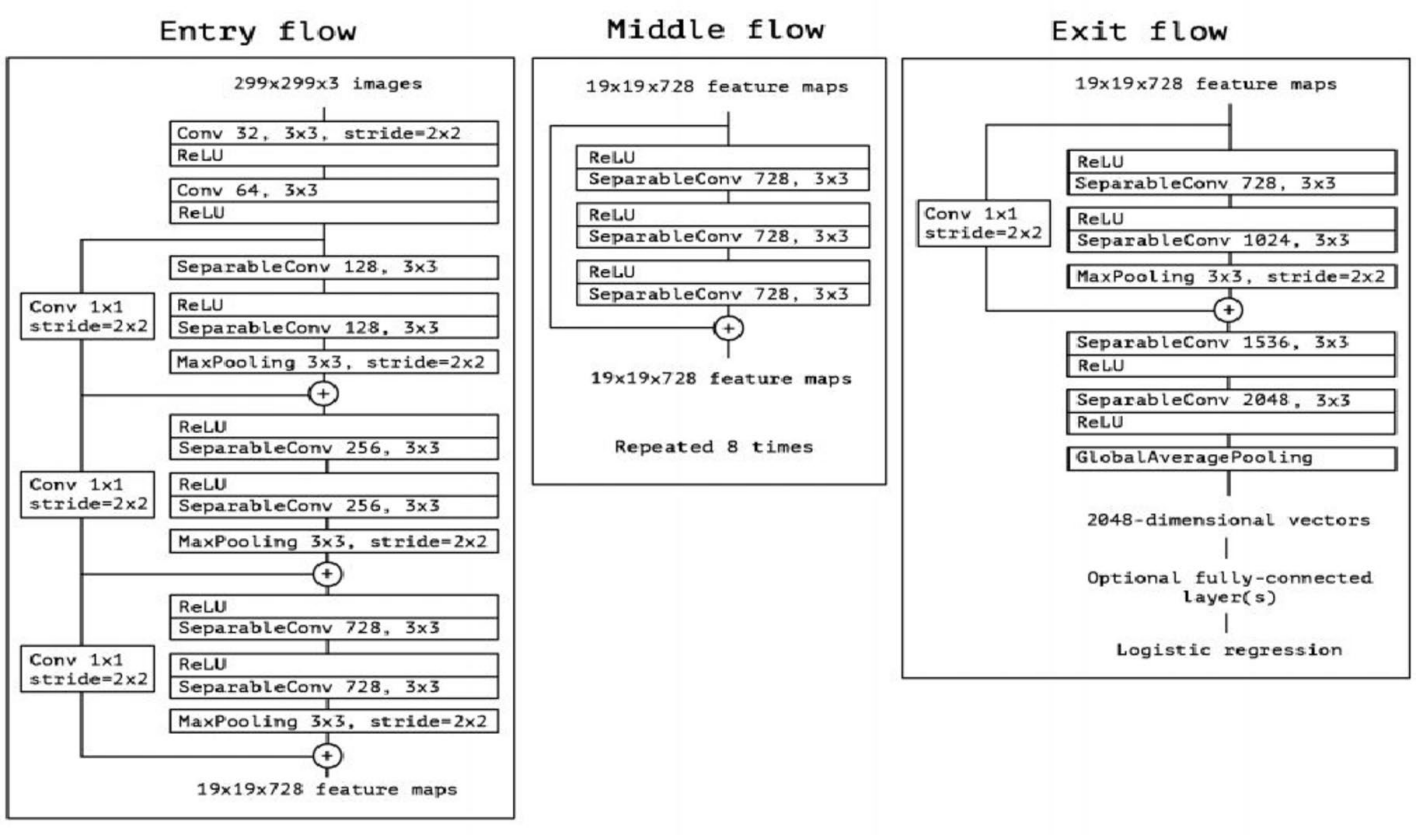
The architecture of Xception.
8. *VGG19*: It contains a total of 19 layers: 16 convolutional and 3 fully connected layers. This deep architecture endows the network with the ability to learn complex features of image data. The uniform architecture consists of stacks of convolutional layers with 3 × 3 convolution filters and 2 × 2 max-pooling layers. This simplicity makes it easy to train and understand. VGG19 comes in several configurations with varying depths, including VGG11, VGG13, VGG16, and VGG19. Thus, the advantage of this is flexibility in the selection of the right architecture for a given task and computation resources size. VGG19 is relatively easy to understand and implement because of its uniform architecture. VGG19 achieved the best performance in various image classification benchmarks. One can take the pre-trained model of VGG19 for the purpose of transfer learning, which is going to enhance the performance of smaller sets significantly.
  *Architecture*: VGG19, a deep network with 19 layers which is represented in Fig. 11.
  *Accuracy*: Achieved an accuracy of 93.5%.
  *Comments*: VGG19 performed well, with accuracy similar to other competitive models.
  **Fig. 11:**
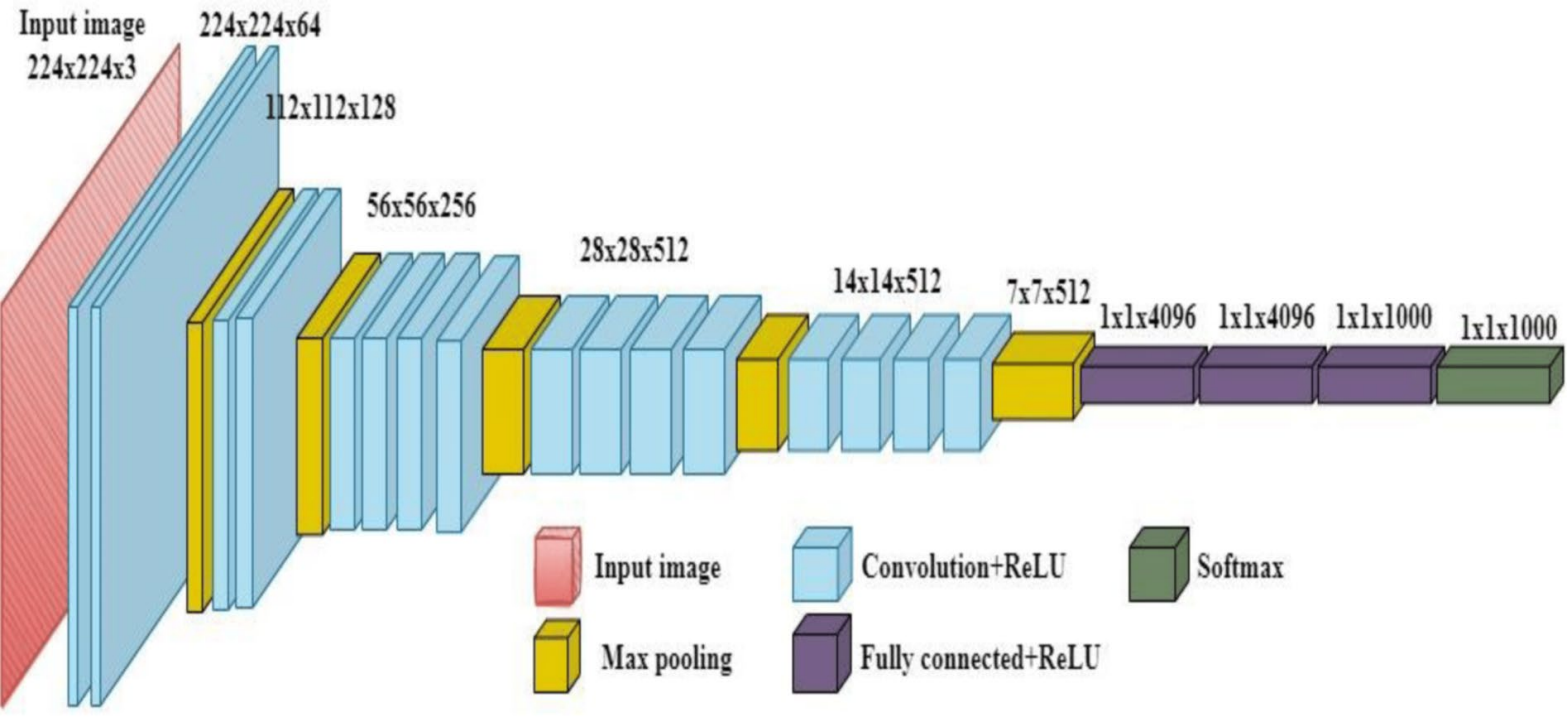
The architecture of VGG19.

## Discussion

ResNetRS50 works better for the detection of blood cancer compared to other models, such as AlexNet and VGG19, can be described as the reason for its architecture. The various reasons are as follows:

- *Residual connections*: ResNetRS50 employs residual connections that allow the network to learn residual functions instead, which makes it easier to train a deep network without suffering from the vanishing gradient problem.
- *Deeper architecture*: It has a deeper architecture than AlexNet and VGG19, thus it can represent more intrinsic features and complex representations of image data. For such reasons, this is very important when classifying blood cancer images, as they usually have very small differences that shallower models cannot detect.
- *RS blocks*: In the ResNetRS50 network, the RSidual Squeeze blocks are employed for their efficacy in the performance of making the network both more efficient and much more accurate. While it achieves a reduced computational cost with these blocks in place, the performance remains high.
- *Transfer learning*: ResNetRS50 can be combined with transfer learning. That means the model is pre-trained on a large-scale dataset, such as ImageNet, and fine-tuned on blood cancer datasets. That may yield very good improvements for the case where there is not enough blood cancer data.

ResNetRS50 offers a strong balance of computational efficiency and scalability; due to some reasons like Computational Efficiency, Scalability, thus proving to be a good choice for real-time applications in clinical settings compared to ConvNext and Inception_v3.

- *Computational efficiency*:
  - *Residual connections*: ResNetRS50 is given residual connections to ensure that even though the networks are far deeper, the vanishing gradient problem does not occur. But this tends to raise the bar when it comes to efficiency in performance.
  - *RS Blocks*: The building blocks, RS, which represent Residual Squeeze, were adopted in ResNetRS50 to reduce the computational cost without any loss of accuracy.
- *Scalability*.
  - *Flexibility*: Easy adaptation of ResNetRS50 to various hardware and task requirements allows ResNetRS50 to apply to a wide range of clinical settings.
  - *Efficient* means that it could run from a high-performance server down to an embedded system.

While ConvNext is quite efficient, it may be somewhat more computationally intense for some tasks than ResNetRS50. Though Inception_v3 is efficient, it does stress computational resources, especially for higher models.

Class imbalance can be handled well through several techniques that range from Data Augmentation to Cost-sensitive Learning using Transfer Learning and combining multiple models when it comes to classifying blood cancer into subtypes that are rare. This can be further improved by training multiple models differing in hyperparameters or data augmentation techniques and combining their predictions.

Also, we delve into a comprehensive discussion of the results obtained from the deployment and evaluation of the ResNetRS50 and RegNetX016 models for cancer detection. The discussion encompasses the models’ performance, their relative strengths and weaknesses, and their potential implications in the field of medical image analysis.

### Model performance

The performance of the ResNetRS50 and RegNetX016 models in cancer detection is a critical aspect of this research. Both models demonstrated commendable accuracy, precision, recall, and F1 scores on the same size of dataset CNMC. However, it is important to delve into specific performance metrics to gain a deeper understanding of their capabilities (refer to Table 1).

**Table 1:** Accuracy classified based on the same dataset CNMC.

Algorithm	Accuracy	Recall	F1 Score
ResNetRS50	97%	99%	98%
RegNetX016	96.4%	99%	98%
AlexNet	87.9%	90%	88%
Convnext	94.2%	94.6%	95%
EfficientNet	93.0%	88%	91%
Inception_v3	92.7%	88%	91%
Xception	91%	89%	92%
VGG19	93.5%	93.6%	93.3%

**Fig. 12:**
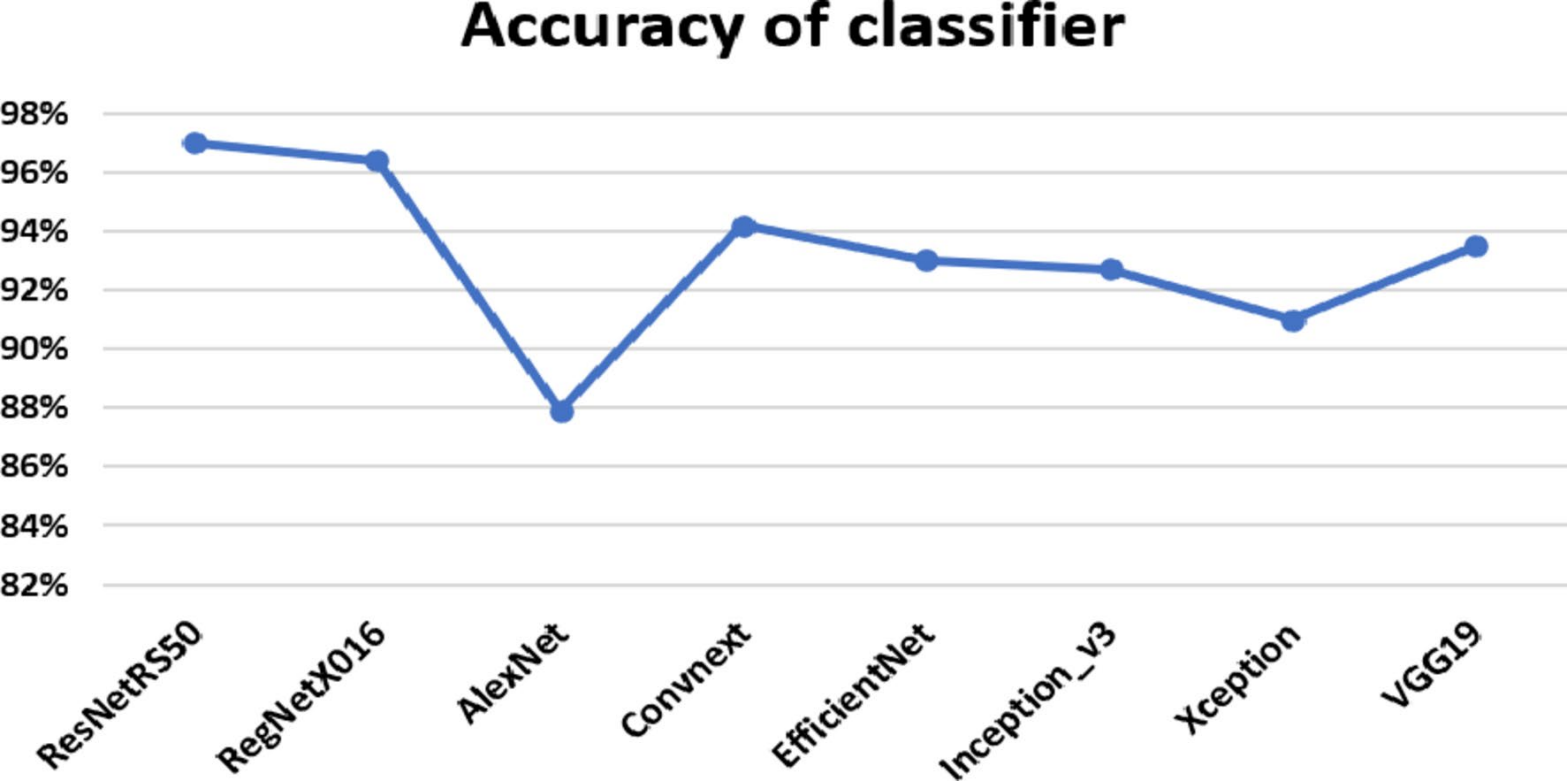
Accuracy of classifier.

**Fig. 13:**
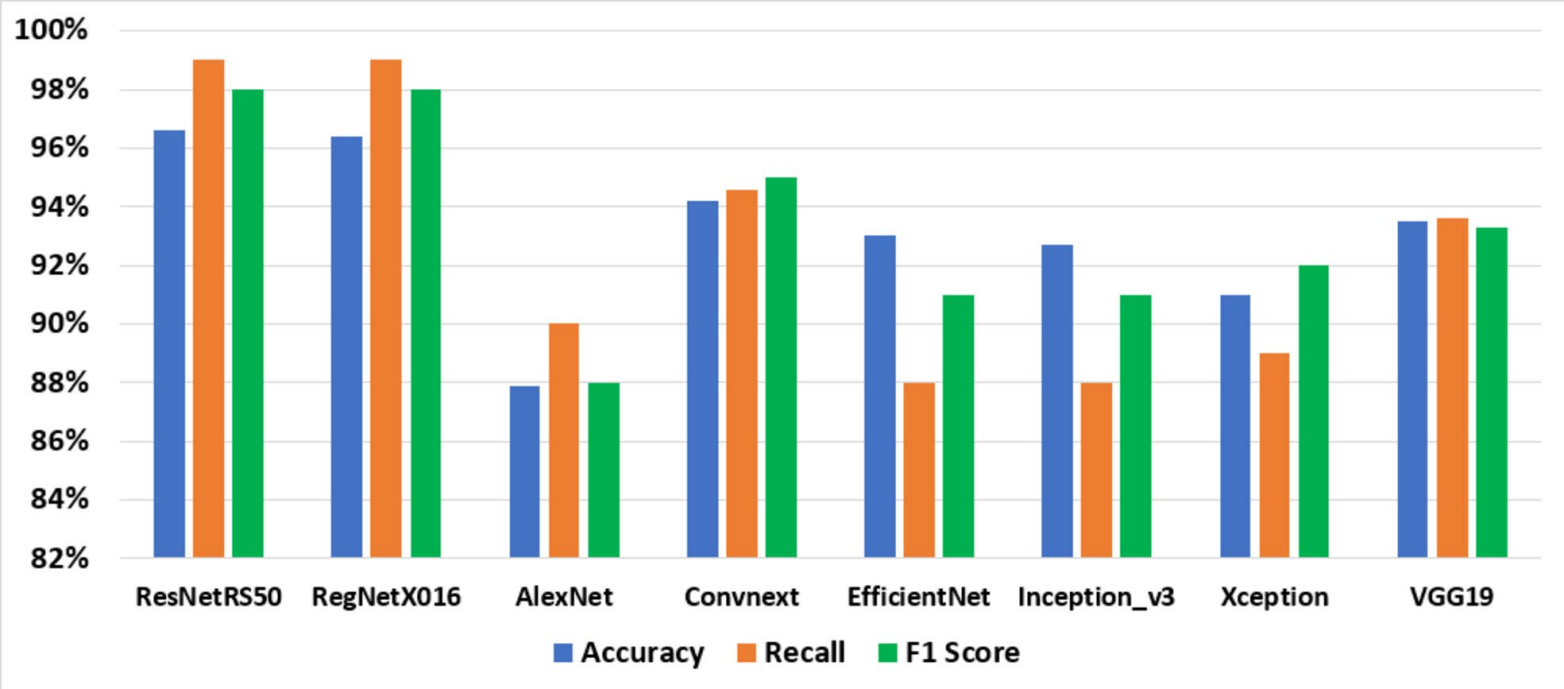
Accuracy of classifier based on accuracy, recall, and F1 scores.

Figures 12 and 13; Table 1 show, Accuracy was calculated for ResNetRS50, RegNetX016, AlexNet, EfficientNet, Inception_v3, Xception, VGG19 and Convnext, by using deep learning. It was found that the best percentage of Training set is 70% and Testing Set 30% at ResNetRS50 algorithm because it gave us the highest Accuracy, (97%). This indicates their ability to classify medical images correctly.

### Confusion matrix

This section explains the plot confusion matrix function, which visualizes the confusion matrix for classification results. The plot confusion matrix function generates a visual representation of the confusion matrix, which is a table used to describe the performance of a classification model.

The Purpose: To create a heatmap of the confusion matrix for easy interpretation of model classification accuracy.

**Fig. 14:**
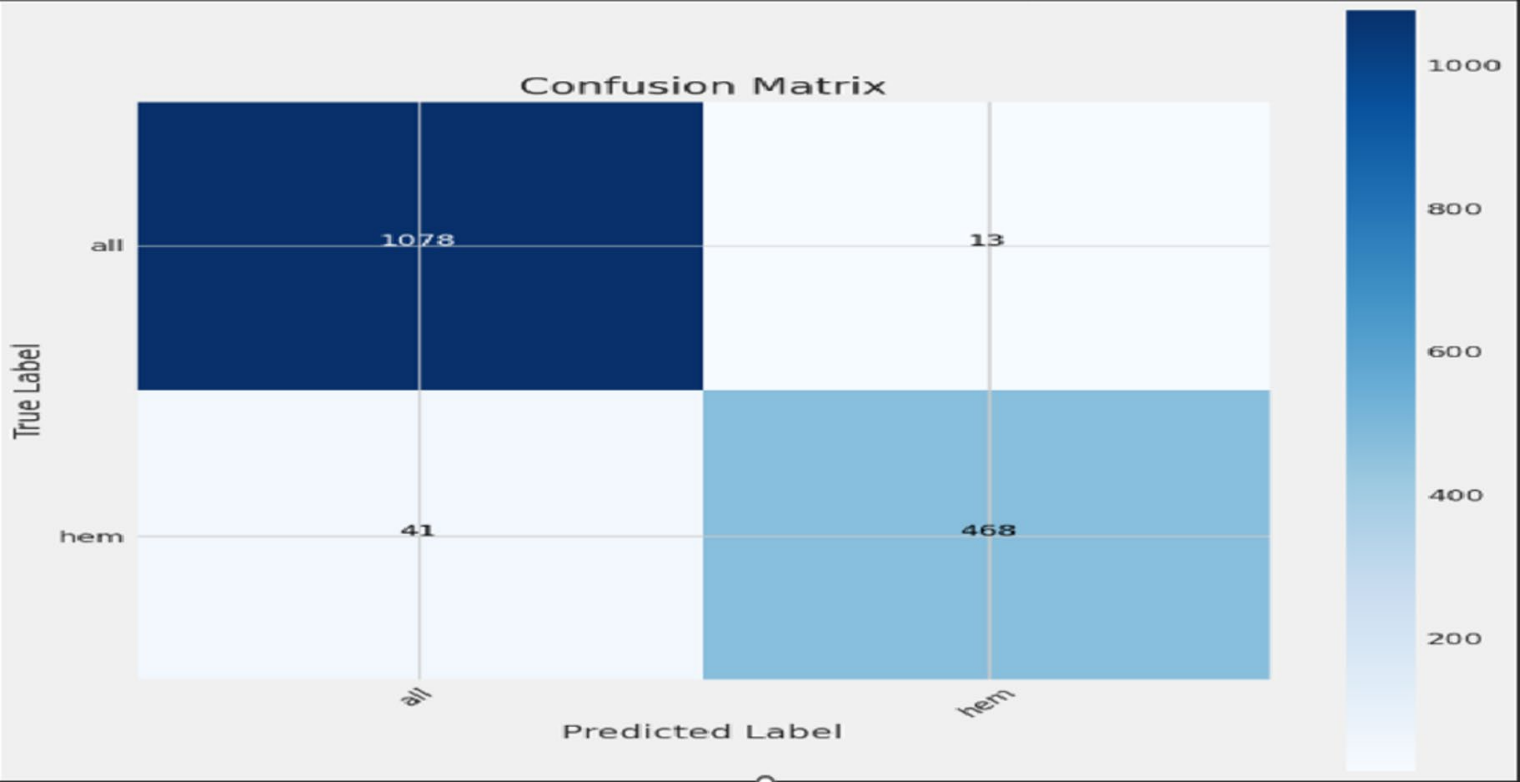
Confusion matrix for RegNetX016.

Figure 14 shows the explanation of the plot confusion matrix function, which visualizes the confusion matrix for classification results. The matrix shows that your model correctly predicted 1078 “all” samples and 468 “hem” samples. It also shows that your model incorrectly predicted 13 “all” samples as “hem” and 41 “hem” samples as “all”.

**Fig. 15:**
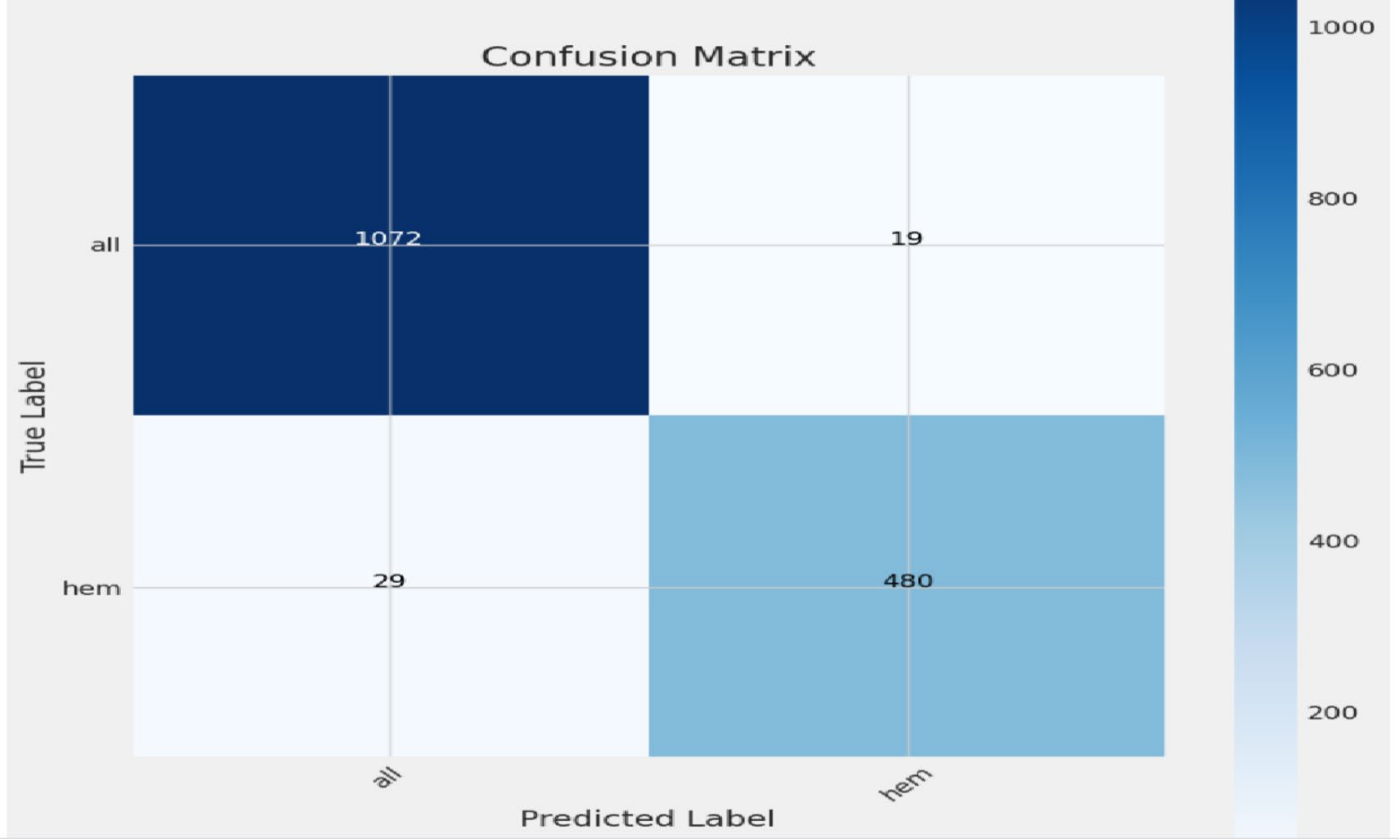
Confusion matrix for ResNetRS50.

Figure 15 shows the explanation of the plot confusion matrix function, which visualizes the confusion matrix for classification results. The matrix shows that your model correctly predicted 1072 “all” samples and 480 “hem” samples. It also shows that your model incorrectly predicted 19 “all” samples as “hem” and 29 “hem” samples as “all”.

## Clinical relevance

The clinical relevance of the ResNetRS50 and RegNetX016 models in a healthcare setting is a pivotal consideration, as they directly impact patient care and the medical community. The models’ high accuracy and performance hold substantial promise for various aspects of cancer diagnosis and treatment.

### Automation of diagnosis

One of the primary clinical benefits of these models is their potential to automate the cancer diagnosis process. The remarkable accuracy achieved in leukemia detection signifies a breakthrough in leveraging artificial intelligence to assist medical professionals. With automation, the burden on healthcare practitioners can be significantly reduced, enabling them to focus more on patient care and decision-making rather than manual image analysis.

Automated diagnosis streamlines the workflow in medical settings, offering several advantages:

A. *Efficiency*: Rapid and accurate diagnosis is crucial in cancer treatment. Automation accelerates the diagnostic process, particularly in scenarios where timely intervention is paramount.
B. *Consistency*: The models provide consistent results, eliminating the variability associated with human interpretation. This is particularly valuable in reducing diagnostic errors.
C. *Scalability*: These models can be deployed across healthcare institutions, ensuring a standardized and high-quality diagnostic process.

### Early detection

A fundamental aspect of clinical relevance lies in the models’ potential to contribute to early cancer detection. As early diagnosis is an indispensable factor in improving patient outcomes and survival rates. Both ResNetRS50 and RegNetX016 demonstrated a remarkable ability to detect cancer, particularly in its nascent stages.

Early detection offers several significant benefits:

A. *Improved treatment*: Identifying cancer at an early stage often leads to more effective treatment options and better prognoses for patients.
B. *Reduced treatment costs*: Earlier diagnosis can reduce the overall cost of cancer treatment, as less aggressive interventions may be required.
C. *Enhanced patient experience*: Early diagnosis can alleviate the physical and emotional burden on patients and their families.

## Challenges and future work

### Ethical considerations

While we have conducted an analysis of the main potential biases existing in the dataset, consideration of ethics in depth is yet to be taken for the purpose of ensuring nondiscriminatory model impact. This will be further refined with the aid of experts in ethics and healthcare.

### Data size and diversity

Increasing dataset size and diversity will make the models robust. The future work shall be done on increasing the dataset with all kinds of demographics and stages of the disease.

### Hyperparameter optimization

Advanced hyperparameter optimization has the real potential for these models. More research is required in methods of hyperparameter optimization to perform model performance tuning.

### Clinical validation

Clinical validation will be necessary to estimate more realistically its performance in a real clinical setup. Cooperation with professionals and institutions should facilitate clinical studies that guarantee safety and effectiveness in patient care.

### Interpretable AI

Methods to explain model decisions will increase trust and facilitate translation into clinical practice.

### Continued model development

The model development process should proceed further. Possibly, future research may work on newer techniques apart from keeping pace with the recent changes happening in the field of deep learning and analysis of medical images.

### Adverse event detection

Further work will be done to enhance the capability of the models in detecting the occurrence of adverse events during treatment for cancer, which will contribute to better patient care through early intervention.

### Global access

Global access needs adaptation of the models according to the diverse resources and infrastructure of the different healthcare settings.

## Conclusion

The ResNetRS50 and RegNetX016 have indeed shown great clinical promise for blood cancer diagnosis automation, hence leading to better patient outcomes with potentially fewer mortality rates. However, these need careful consideration of the ethical implications so that deployment is done in a manner that is equitable and beneficial. Further research will be necessary to refine these models into real-world clinical applications. From the analysis, ResNetRS50 scored an accuracy rate of 97% against 3% of error rates. This will therefore mean that ResNetRS50 has a high possibility of becoming the helper for blood cancer diagnosis.

## Data Availability

https://www.cancerimagingarchive.net/collection/c-nmc-2019/.
